# IUC26693-91 Cholesterol homeostasis and immune-inflammatory profiles in patients with mRCC receiving immunotherapy

**DOI:** 10.1093/oncolo/oyag286.027

**Published:** 2026-08-21

**Authors:** P Tuttobene, M Maffezzoli, G Leali, M Pluchino, E Favari, G Mazzaschi, F Pecci, M Tiseo, M Di Maio, S Buti

**Affiliations:** Department of Medicine and Surgery, University of Parma, Parma, Italy - Italy; Medical Oncology Unit, University Hospital of Parma, Parma, Italy - Italy; Department of Medicine and Surgery, University of Parma, Parma, Italy - Italy; Department of Medicine and Surgery, University of Parma, Parma, Italy; Medical Oncology Unit, University Hospital of Parma, Parma, Italy - Italy; Department of Food and Drug, University of Parma, Parma, Italy - Italy; Department of Medicine and Surgery, University of Parma, Parma, Italy; Medical Oncology Unit, University Hospital of Parma, Parma, Italy - Italy; Department of Medicine and Surgery, University of Parma, Parma, Italy; Medical Oncology Unit, University Hospital of Parma, Parma, Italy - Italy; Department of Medicine and Surgery, University of Parma, Parma, Italy; Medical Oncology Unit, University Hospital of Parma, Parma, Italy - Italy; Medical Oncology 1, AOU Città della Salute e della Scienza di Torino, Turin, Italy - Italy; Department of Medicine and Surgery, University of Parma, Parma, Italy; Medical Oncology Unit, University Hospital of Parma, Parma, Italy - Italy

## Abstract

**Background:**

Cholesterol metabolism may modulate antitumor immunity, influencing response to immune-checkpoint inhibitors (ICIs). We evaluated cholesterol transport-related biomarkers, KIM-1, and inflammatory mediators in metastatic renal cell carcinoma (mRCC) patients treated with first-line ICI-based regimens, to characterize the immune–metabolic profiles.

**Methods:**

LINCHOLM is a prospective, multicentre, observational study. Patients with mRCC receiving ICI-based combinations underwent blood collection at baseline, week 6 and progression. ABCA1- and ABCG1-mediated cholesterol efflux (CE), lipid fractions, and inflammatory mediators were assessed. Primary objective was to investigate associations between baseline cholesterol transporters and inflammatory mediators, and progression-free survival (PFS).

**Results:**

33 patients were included, 84.8% had ECOG PS 0-1. Across worsening IMDC risk groups Apo-A1, total cholesterol, HDL and LDL decreased (all *p*≤0.03), whereas KIM-1, IL-6 and Apo-E increased (all *p*≤0.02). High ABCG1-mediated CE correlated with higher levels of ApoE and IL-1β (all *p*≤0.04). High KIM-1 was associated with poorer ECOG PS, synchronous metastatic disease, and higher NLR, IL-4, IL-6, and IL-10 (all *p*≤0.04), **Figure 1**. High baseline ABCG1-mediated CE, KIM-1, ApoE, NLR, and inflammatory cytokines (IL-4, IL-6, IFN-γ, IL-10, IL-1β, IL-2, and TNF-α) correlated with shorter PFS, **Table 1**. Only ABCG1 remained independently associated with worse PFS at multivariable analysis (HR 10.61, 95%CI 2.35–47.97; *p* = 0.002), although estimates were limited by small sample size.

**Conclusions:**

ABCG1-mediated CE may serve as a prognostic biomarker in mRCC patients receiving first-line ICI-based therapies. The interplay among cholesterol metabolism, KIM-1, and inflammatory mediators suggests a biological profile influencing outcomes and warrants further validation.

**Acknowledgements:**

Project funded by the European Union - NextGenerationEU, Mission 4 Component 1, Unique Project Code (CUP): D53D23013940006

**IUC26693-91 Table 1:** Selected biomarkers significantly associated with PFS.

Variable	Low group, median PFS, months (95% CI)	High group, median PFS, months (95% CI)	*p*-value
**ABCG1**	12.07 (6.81–NE)	1.81 (1.15–NE)	<0.001
**NLR**	16.09 (7.07–NE)	2.57 (1.45–NE)	0.008
**KIM-1**	16.09 (7.53–NE)	2.62 (1.45–NE)	0.002
**ApoE**	12.07 (6.81–NE)	1.51 (0.89–NE)	<0.001
**IL-6**	12.07 (6.78–NE)	1.45 (1.18–NE)	0.009
**IL-1β**	12.07 (6.81–NE)	1.22 (1.15–NE)	0.002
**TNF-α**	12.07 (6.81–NE)	1.64 (1.25–NE)	0.002

**IUC26693-91 Figure 1:**
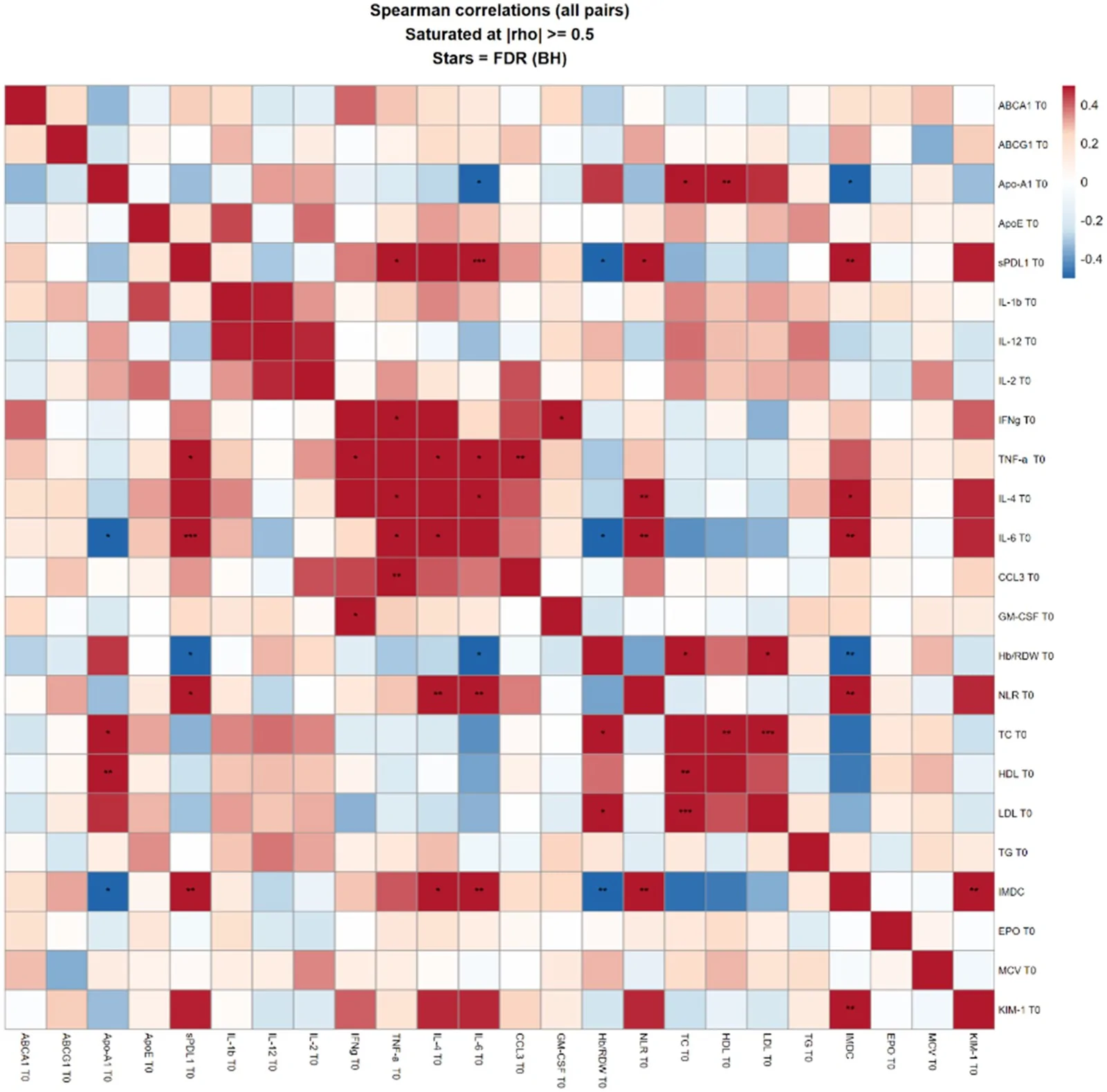
Heatmap of correlations between baseline biomarkers.

